# Everybody's Page

**Published:** 1889-04-13

**Authors:** 


### Everybody's Page.

Frank Buckland declared that the flesh of boa-constrictor was much more delicate than veal.

In 1887, 4,450lb. of rosewater and other perfumes, of the value of £55,000, were exported from Phippopolis.

Eggs can be preserved fresh for three months by carefully oiling them all over with a soft brush, and packing them in a jar, with plenty of bran between each layer.

Aristotle says: It is useless to try to keep a young creature quiet, and just as a rattle is the proper thing for babies, so musical instruction is the proper rattle for older children.

The Indians on the Pacific Coasts of America looked upon an invasion of grasshoppers that occurred in 1875 as a special favour of the Great Spirit, who thus sent them food enough to lay up a provision for several years to come.

The Liebig Extract of Beef Company has £3,000,000 invested at Fray Bentos, a little town in Uruguay, where it kills perhaps half a million head of cattle every year. The annual sale of the Liebig Company's extract now exceeds eight million jars.

Castor oil seeds, when eaten, are distinctly poisonous, causing internal inflammation with obstruction of the bile-duct. Ten seeds are enough to kill a man, but Christison once had a fatal case when only three seeds had been swallowed, and on the other hand a case is known to have recovered when seventeen seeds had been taken.

In Roumelia, where roses grow by the million, the leaves are made into jam. It hardly seems right to consume flowers in that fashion, and certainly the crystallised violets we buy here are not worth the sense of desecration one feels in eating them; but the rose preserve, or "dulchatz," as such dainties are called in the East, is by no means so insipid as might be expected. Sweet it is, but it has a sharp after taste that is very refreshing.

Cock-fighting used to be a recognised sport for schoolboys. An item in Sir James Mackintosh's bill at Fortrose school, drawn up by the master, was "1776-7. To cocks'-fight dues for two years, 2s. 6d. each, 5s." Hugh Miller, the geologist, also narrates in "My Schools and Schoolmasters," the excitement caused by the yearly cock-fight in Cromarty Grammar School. The boys had two holidays before the cock-fight to collect and prepare the cocks, and for weeks afterwards their conversation would consist largely of anecdotes of the courage and ferocity shown by the miserable birds.

The *American Druggist* tells of a horse that wears spectacles. The farmer that owned him having come to the conclusion, from various symptoms, that the animal was short-sighted, got an oculist to take the necessary measurements, and had a pair of spectacles manufactured for him. They are made to fasten firmly into the headstall, so that they do not shake out of place. At first, the horse appeared startled by this addition to his harness, but he soon got used to his glasses, and liked them. "In fact," says his owner, "when I turn him out to pasture he feels uneasy and uncomfortable without his goggles, and last Sunday he hung around the barn and whinnied so plaintively that I put the headstall and goggles on him, and he was so glad that he rubbed my shoulders with his nose. Then he kicked up his heels, and danced down to the pasture." He could see what he was going to eat then.

Rousseau defined suicide as "a furtive and shameful death; a theft from the human race."

The peculiar fragrance of Russian leather is due to the oil or tar derived from birchwood, which is used in tanning it.

"What becomes of the old moons, papa?" asked the enquiring child. "The old moons, my son?" answered the parent, "Why, they die of newmonia, to be sure."

Prof. Poel, of St. Petersburg, found that the cod-liver oil sold by one druggist contained 50 per cent. of petroleum. The adulterated article could not be detected either by taste, smell, or appearance.

In 1702 Solomon Hottinger, of Zurich, expressed the opinion that the medical waters of Baden were of undoubted efficacy, "because," he says, "on that point the experience of every day and every year are decisive."

A new sedative, called boldoin, has been discovered by M. Juranville. It is derived from boldo leaves, and is said to be more powerful as a narcotic than either opium or chloral, while a pretty large dose can be administered to an invalid without ill effects.

The nurse had shown Bobby the new baby, and asked him what he thought of it. He considered the matter for a moment, and then answered, critically, "I suppose it's nice enough, what there is of it, but I wish it had been a parrot." Was Bobby an unconscious believer in the transmigration of souls?

Although so much is said about railway accidents, they really are very rare things. Mark Twain once compared the mortality statistics of a few large towns with those of deaths due, directly or indirectly, to injury from railway travelling, and came to the conclusion that if he wanted a long life he should spend it in the trains. As a matter of fact, only one traveller in every ten millions is killed by a railway accident.

In the good old days of smuggling, dogs used to prove themselves the superiors of men in the art, when it was a question of carrying light goods over an excise-guarded frontier. The dog was first taught the route he was to take. To this end he was kept starving for twenty-four hours, then led to his destination, where food and caresses in plenty awaited him. This impressed on his mind the idea of returning to the place where he had been so well treated. On his subsequent journeys, he wore a coat which had originally belonged to a larger dog, and between this and his own skin was packed the lace or other contraband goods he was to convey. At the present day, dogs are believed to be the means of carrying on a brisk smuggling trade in cigars between Gibraltar and Spain.

When a man wants to get drunk, he will take anything in the shape of an intoxicant. The clergymen in the North of Ireland have resolved to petition Parliament to make some regulation limiting the sale of ether, as the habit of ether drinking is spreading among the people. The merit of ether is its cheapness; a man can get enough for a penny to stupefy him to his heart's content. In Edinburgh there have been several cases of drunkenness from methylated spirit, and some druggists have got into trouble for selling the spirit with the knowledge that it was to be used, either openly or as an adulterant, for drinking purposes. The energy of Parliament in trying to make men virtuous seems quite to be surpassed by the ingenuity displayed by the viciously disposed in evading restrictions.

